# Functional Characterization of *PALB2* Variants of Uncertain Significance: Toward Cancer Risk and Therapy Response Prediction

**DOI:** 10.3389/fmolb.2020.00169

**Published:** 2020-09-16

**Authors:** Rick A. C. M. Boonen, Maaike P. G. Vreeswijk, Haico van Attikum

**Affiliations:** Department of Human Genetics, Leiden University Medical Center, Leiden, Netherlands

**Keywords:** breast cancer, variant of uncertain significance (VUS), PALB2, DNA repair, homologous recombination (HR), PARP inhibitor (PARPi)

## Abstract

In recent years it has become clear that pathogenic variants in *PALB2* are associated with a high risk for breast, ovarian and pancreatic cancer. However, the clinical relevance of variants of uncertain significance (VUS) in *PALB2*, which are increasingly identified through clinical genetic testing, is unclear. Here we review recent advances in the functional characterization of VUS in *PALB2*. A combination of assays has been used to assess the impact of *PALB2* VUS on its function in DNA repair by homologous recombination, cell cycle regulation and the control of cellular levels of reactive oxygen species (ROS). We discuss the outcome of this comprehensive analysis of *PALB2* VUS, which showed that VUS in PALB2’s Coiled-Coil (CC) domain can impair the interaction with BRCA1, whereas VUS in its WD40 domain affect PALB2 protein stability. Accordingly, the CC and WD40 domains of PALB2 represent hotspots for variants that impair PALB2 protein function. We also provide a future perspective on the high-throughput analysis of VUS in *PALB2*, as well as the functional characterization of variants that affect *PALB2* RNA splicing. Finally, we discuss how results from these functional assays can be valuable for predicting cancer risk and responsiveness to cancer therapy, such as treatment with PARP inhibitor- or platinum-based chemotherapy.

## PALB2 Is Essential for DNA Repair by Homologous Recombination

The integrity of our genome is relentlessly challenged by exogenous and endogenous insults that can induce DNA damage. To respond to such genotoxic threats, cells have evolved a number of DNA damage signaling and repair mechanisms, jointly known as the DNA damage response (DDR). The DDR is able to handle a myriad of DNA damages of which DNA double strand breaks (DSBs) are considered among the most deleterious to the cell. Human cells possess at least five pathways for DSB repair: canonical non-homologous end joining (c-NHEJ), alternative non-homologous end-joining (a-NHEJ), single-strand annealing (SSA), break-induced replication (BIR), and homologous recombination (HR) (Ciccia and Elledge, 2010; Chapman et al., 2012). c-NHEJ is the predominant DSB repair pathway in human cells and complete loss-of-function (LOF) is likely to drive cell death due to an unreasonably high DSB burden (Sishc and Davis, 2017). In case c-NHEJ fails or is inappropriate, HR is probably the most frequently used alternative pathway for DSB repair. However, while c-NHEJ is active throughout the whole cell cycle, HR is restricted to late S/G2 phase as it relies on the presence of an undamaged sister chromatid to act as a template for error free repair (Hustedt and Durocher, 2016). During HR, BRCA1 inhibits 53BP1 from interacting with the chromatin near the broken DNA ends (Chapman et al., 2012; Densham et al., 2016). This permits extensive end-resection of the break by endo- and exonucleases such as MRE11, CtIP, DNA2, and EXO1, yielding 3′-single-stranded (ss) DNA overhangs that counter Ku loading and further promote DSB repair by HR (Marini et al., 2019). Following resection, the 3′-ssDNA tails become coated by the RPA heterotrimer (Symington, 2016). Subsequently, BRCA1, PALB2 and BRCA2 sequentially accumulate on the processed ssDNA to promote error-free repair of DSBs.

**FIGURE 1:**
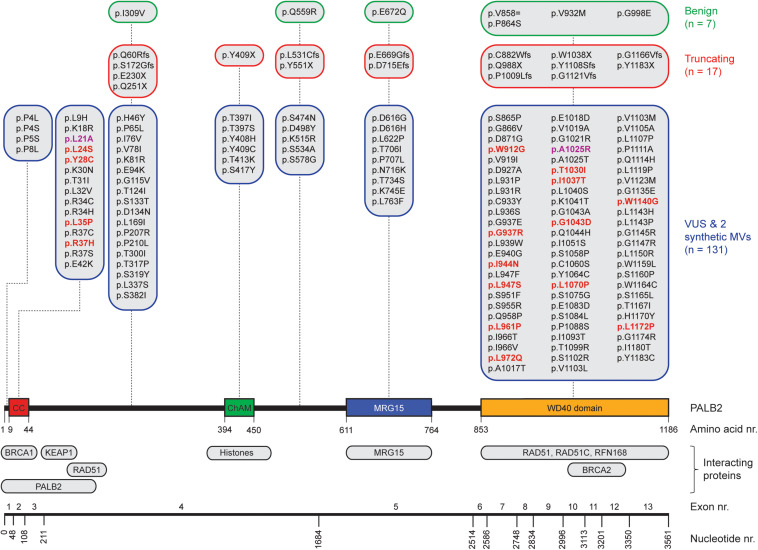
Schematic representation of *PALB2* variants, functional domains, interacting proteins, and exons. The nucleotide numbers refer to the last nucleotide of each exon in *PALB2* cDNA (NM_024675.3). The amino acid numbers are shown to specify the evolutionarily conserved functional domains of PALB2; Coiled-coil (CC) (Tischkowitz et al., 2007; Sy et al., 2009b; Zhang et al., 2009a,b), Chromatin-Association Motif (ChAM) (Bleuyard et al., 2012), MORF-Related Gene on chromosome 15 (MRG15) binding domain (Sy et al., 2009a) and WD40 domain (Xia et al., 2006; Tischkowitz et al., 2007). PALB2-interacting proteins are depicted underneath their respective PALB2 interacting domain/regions. All *PALB2* genetic variants from five functional studies (Park et al., 2014; Foo et al., 2017; Boonen et al., 2019; Rodrigue et al., 2019; Wiltshire et al., 2019) are shown and categorized per (functional) domain as benign (green framed sections), truncating (red framed sections), or VUS and synthetic missense variants (MVs) (blue framed sections) based on ClinVar. All functionally damaging *PALB2* VUS with an HR efficiency < 50% compared to wild type *PALB2* in at least one functional assay are highlighted in red. The two damaging synthetic MVs are highlighted in purple.

PALB2 is crucial herein as it mediates PALB2-BRCA1/2-RAD51 complex formation. That is, PALB2’s N-terminal Coiled-Coil (CC) domain is required for interaction with BRCA1, whereas its C-terminal WD40 domain mediates the interaction with BRCA2 (Figure 1) (Xia et al., 2006; Sy et al., 2009b; Zhang et al., 2009a,b; Prakash et al., 2015). BRCA2 possesses eight highly conserved BRC repeats and a carboxy-terminal region that have been shown to bind RAD51 (Bignell et al., 1997; Wong et al., 1997; Esashi et al., 2005). This interaction allows BRCA2 to promote HR by facilitating the replacement of RPA with the RAD51 recombinase and by stabilizing the ensuing RAD51-ssDNA filaments through blockage of ATP hydrolysis (Jensen et al., 2010). Additionally, through its WD40 domain, PALB2 also interacts with the C-terminal PALB2-Interacting Domain (PID) of the RNF168 ubiquitin E3 ligase. RNF168 contains a ubiquitin-interacting motif (UIM) that allows binding of RNF168-bound PALB2 to ubiquitylated chromatin at DSBs, thereby facilitating RAD51 filament formation and HR (Luijsterburg et al., 2017). Alternatively, more recent studies suggested that RNF168 may facilitate PALB2-mediated RAD51 loading independently of BRCA1, by showing that abrogation of RNF168 activity in BRCA1-compromised cells dramatically elevated genome instability rates (Zong et al., 2019; Callen et al., 2020). Thus, it is apparent that RAD51 loading during HR, regardless of its dependency on BRCA1, RNF168, or both, requires the action of PALB2.

## Genetic Variants in *PALB2* and Their Association With Cancer

Recent analysis of a metastatic pan-cancer cohort of 3504 patients, employing a strategy that relies on the presence of specific mutational footprints which are characteristic of a deficiency in HR (Nik-Zainal et al., 2016; Polak et al., 2017), revealed that mutational inactivation of the *BRCA1*, *BRCA2*, and *PALB2* genes was the most common genetic cause of the observed HR signatures (Nguyen et al., 2020), indicative of their important role in tumor suppression. Indeed, for *BRCA1* and *BRCA2*, monoallelic LOF variants present in the germline can result in a nearly tenfold increased lifetime risk of developing breast cancer (Antoniou et al., 2003, 2014), whereas bi-allelic LOF variants cause Fanconi anemia (FA) (Howlett et al., 2002; Sawyer et al., 2015). The *PALB2* gene, which is located on chromosome 16p12.2, comprises 13 exons and encodes a protein of 1186-amino acids (Figure 1), was identified in 2006 as an important BRCA2-interacting protein (Xia et al., 2006; Tischkowitz and Xia, 2010). As it has now been established that LOF variants in *PALB2* convey a similarly high risk for breast cancer as *BRCA2* LOF variants (Antoniou et al., 2003, 2014; Couch et al., 2017), *PALB2* has become widely included in breast cancer clinical genetics practice. Consequently, a large number of people have already undergone genetic testing of *PALB2* to identify variants that may increase the risk of breast cancer susceptibility. Meanwhile, truncating *PALB2* variants have also been shown to be associated with an increased risk of familial ovarian and pancreatic cancer (Jones et al., 2009; Slater et al., 2010; Hofstatter et al., 2011; Hu et al., 2018; Yang et al., 2020).

In contrast to truncating variants in *PALB2*, which are known to be deleterious to protein function, the impact of most missense variants is often unclear. Generally, assessment of pathogenicity of such variants of uncertain significance (VUS) would rely mostly on *in silico* analysis, co-segregation of the variant with cancer, co-occurrence with pathogenic *PALB2* variants and family history of cancer. However, for the majority of VUS, this information is not available and hence the associated cancer risk is unknown. To extend the utility of *PALB2* genetic test results, additional methods for interpreting VUS are therefore urgently required. Accordingly, recent independent studies have developed functional assays to determine the functional impact of a large number of *PALB2* VUS (Figure 1). Here we review this comprehensive analysis, which highlights the CC and WD40 domains of PALB2 as hotspots for variants that impair its function in HR and cell cycle checkpoint regulation. Finally, we also highlight the value of this functional analysis in predicting the associated cancer risk and therapy response for VUS in *PALB2.*

## A Comprehensive Functional Analysis of VUS in *PALB2*

**FIGURE 2:**
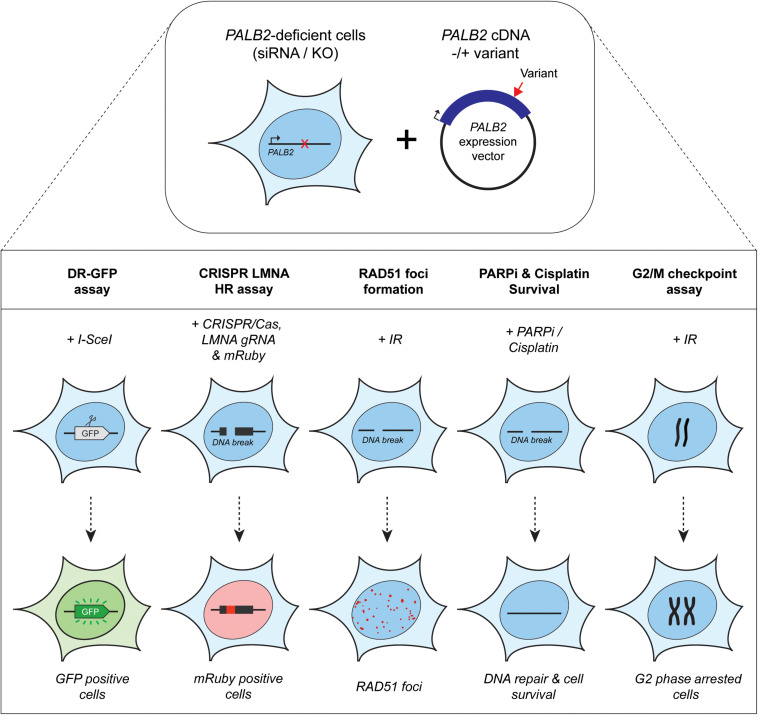
Overview of the functional assays used for the functional characterization of *PALB2* genetic variants. Either *Palb2* KO mouse cells or *PALB2* siRNA-depleted human cells were complemented by expressing human *PALB2* (siRNA-resistant) cDNA, without or with a variant. *PALB2* deficiency is indicated with a red cross, whereas a red arrow marks the position of a variant in the *PALB2* cDNA. Complementation was either by transient (B400 mouse cells or human cell lines) or stable expression (mES cells) **(top section)**. *PALB2* complemented cells were subjected to multiple cell-based functional assays **(bottom section)**. The functional assays determine in a quantitative manner: (1) homology-directed repair of an I-*Sce*I–induced DSB in DR-GFP, which results in the restoration of a functional *GFP* gene whose expression can be monitored by fluorescence-activated cell sorting (FACS), (2) HR-mediated integration of mRuby into the *LMNA* A/C locus (LMNA) at a break site induced by CRISPR/Cas9, (3) the formation of IR-induced RAD51 foci, which is PALB2 dependent and provides a measure for the HR efficiency, (4) sensitivity to PARPi or cisplatin treatment, which leads to cell killing when HR is impaired, (5) G2/M checkpoint maintenance after extensive DNA damage, which is dependent on PALB2-mediated HR. Deficiency in PALB2 results in the progression of cells from G2-phase into M-phase. Consequently, the mitotic fraction represents a measure for the functional impact of *PALB2* variants.

Assays using HR as a read-out have emerged as the standard for the functional characterisation of VUS in *BRCA1* and *BRCA2* (Bouwman et al., 2013; Woods et al., 2016; Shimelis et al., 2017; Starita et al., 2018; Mesman et al., 2019). More recently, VUS in *PALB2* have also been characterized in a similar manner (Figures 1, 2) (Park et al., 2014; Foo et al., 2017; Boonen et al., 2019; Rodrigue et al., 2019; Wiltshire et al., 2019). To identify variants that impact HR, the well described DR-GFP reporter, as well as the more recently introduced CRISPR-LMNA HR assay were used (Kass et al., 2013; Ducy et al., 2019). These assays rely on HR-mediated repair of a non-functional GFP gene and HR-mediated integration of a fluorescence marker at the *LMNA* locus, respectively (Figure 2). Furthermore, PALB2 function was assessed by exposing cells that express a *PALB2* variant to PARP inhibitor (PARPi) or cisplatin (Figure 2). Catalytic inhibition of PARP1 “traps” PARP1 molecules on endogenous ssDNA breaks, resulting in replication fork collapse and DSB formation (Murai et al., 2012). Cisplatin on the other hand, induces ∼90% intra-strand cross-links and ∼5% inter-strand cross-links (ICLs), the latter of which are converted into DSBs through the FA pathway (Deans and West, 2011). Both PARPi- and ICL-induced DSBs are repaired by HR. Consequently, in the absence of HR (e.g., due to PALB2 LOF), PARP-trapping or ICL induction leads to persistent accumulation of DSBs. Such extensive DNA damage often results in cell cycle arrest and apoptosis, and thus reduced proliferation and cell survival. PALB2 LOF is therefore synthetic lethal with PARPi or cisplatin treatment (McCabe et al., 2006; Lord and Ashworth, 2016, 2017). Furthermore, PALB2 is also required for the repair of ionizing radiation-induced DSBs. This phenotype was used as a readout for the functional characterization of several *PALB2* variants, revealing that two variants, p.L939W and p.L1143P, impaired PALB2 function (Park et al., 2014). Lastly, since PALB2 interacts with BRCA1 and BRCA2 to load RAD51 at sites of DSBs, co-immunoprecipitation, recruitment to laser micro-irradiation induced DNA damage, and DNA-damage-induced RAD51 foci formation were among the additional functional readouts to study the impact of *PALB2* variants on HR (Figure 2) (Park et al., 2014; Foo et al., 2017; Boonen et al., 2019; Rodrigue et al., 2019; Wiltshire et al., 2019). A complete overview of all functional assays that were performed by three recent studies is provided in Table 1 (Boonen et al., 2019; Rodrigue et al., 2019; Wiltshire et al., 2019). With the above described functional assays, these studies analyzed a total of 155 different *PALB2* variants (Table 2), comprising 129 VUS, 7 benign variants (as classified by ClinVar) (Landrum et al., 2014), 2 synthetic missense variants with known LOF (Oliver et al., 2009; Sy et al., 2009b) and 17 truncating variants (Figure 1). Sixteen VUS were identified as strongly damaging in at least one assay (i.e., >50% reduced activity compared to WT), all of which were located in the CC or WD40 domain of PALB2 (Figure 1), highlighting the importance of these domains for PALB2’s role HR. In the following sections, we review the different strategies and outcomes of these studies in more depth.

**TABLE 1:** Complete list of functional assays used in three independent studies.

**Study**	**Functional assay**	**Nr. of variants tested (patient derived)**
**Boonen et al., 2019 (70 variants)**	DR-GFP reporter	Complete set
	PARPi sensitivity (proliferation)	Complete set
	PALB2 expression blots	Complete set
	PARPi sensitivity (clonogenic)	8
	Cisplatin sensitivity (proliferation)	18
	RAD51 foci number after IR	5
	RAD51 foci intensity after IR	2
	CRISPR-LMNA HR	5
	G2 > M checkpoint	19
	Recruitment to laser-induced DNA damage tracks	3
	Co-immunoprecipitation	3
**Rodrigue et al., 2019 (47 variants)**	PARPi sensitivity (proliferation)	Complete set
	PALB2 expression blots	Complete set
	RAD51 foci number after IR	18
	RAD51 foci intensity after IR	8
	CRISPR-LMNA HR assay	18
	Recruitment to laser-induced DNA damage tracks	18
	PALB2 cellular localization	18
	Mammalian two-hybrid assay (BRCA1) (1-319)	22
	Mammalian two-hybrid assay (BRCA2) (859-1186)	25
**Wiltshire et al., 2019 (91 variants)**	DR-GFP reporter	Complete set
	PARPi sensitivity (proliferation)	5
	PALB2 expression blots	6
	Cisplatin sensitivity (proliferation)	5
	RAD51 foci number after IR	4
	CRISPR-LMNA HR assay	4
	Recruitment to laser-induced DNA damage tracks	4
	PALB2 cellular localization	4
	Mammalian two-hybrid assay (BRCA1) (1-319)	3
	Mammalian two-hybrid assay (BRCA2) (859–1186)	3
	Cyclohexamide chase	5
	Co-immunoprecipitation	6

**TABLE 2:** Complete list of human *PALB2* variants analyzed in five independent studies.

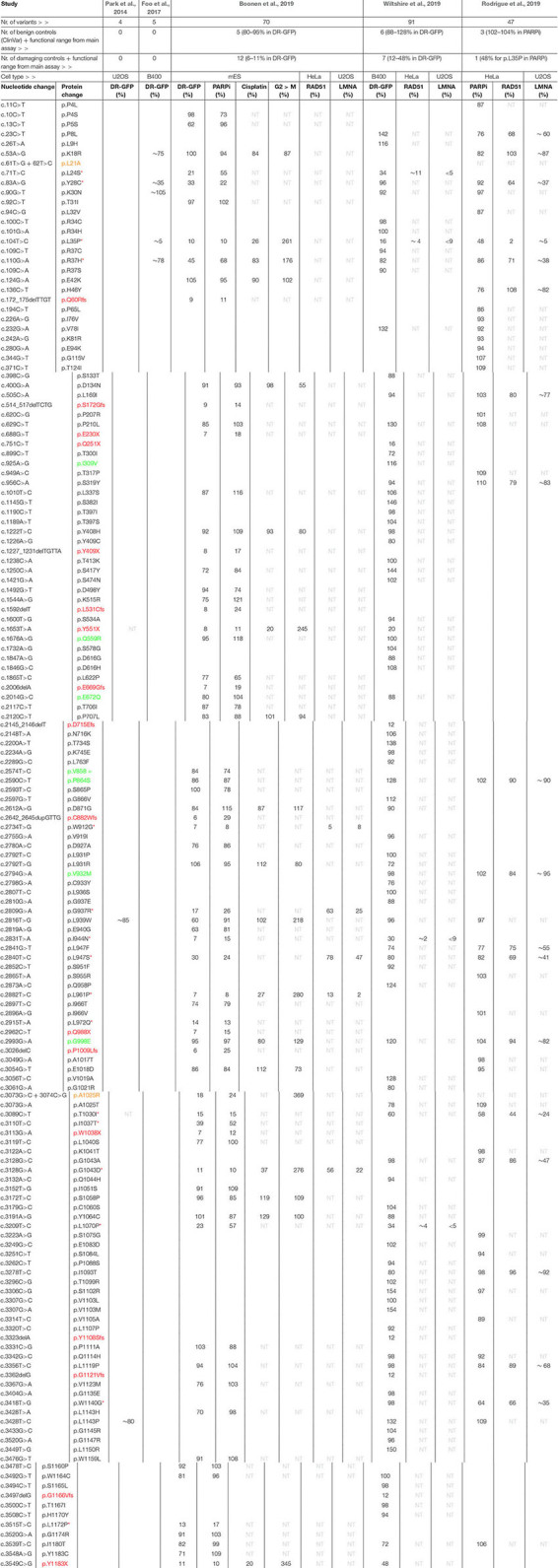

## Functional Characterization of VUS in *PALB2* Using HR as a Read-Out

The largest set of *PALB2* variants, i.e., 84 patient-derived *PALB2* missense variants, was analyzed by Wiltshire and colleagues (Table 2) (Wiltshire et al., 2019). Several truncating variants (p.Q251X, p.Y551X p.D715Efs, p.Y1108Sfs, p.G1121Vfs, p.G1166Vfs, and p.Y1183X) and benign missense variants as classified by ClinVar (p.I309V, p.Q559R, p.E672Q, p.P864S, p.V932M, and p.G998E) were analyzed to validate their functional impact. The assays were mostly performed in *Palb2*-deficient B400 mouse mammary tumor cells with a stably integrated DR-GFP reporter to measure HR. *PALB2* cDNA, with or without a variant, was transiently (over-)expressed in these cells and subsequently the effect on HR was determined. While benign variants had only a moderate or no impact on HR (<12% reduction in HR when compared to WT *PALB2*), all truncating variants strongly impacted HR (>52% reduction in HR when compared to WT *PALB2*). Moreover, four *PALB2* missense variants (p.L24S, p.L35P, p.I944N, and p.L1070P) were identified that strongly disrupted HR (>65% reduction in HR compared to WT *PALB2*). To corroborate their findings, a CRISPR-*LMNA* HR assay (Ducy et al., 2019) was performed in U2OS cells with endogenous *PALB2* depletion by siRNA treatment, followed by transient expression of siRNA-resistant *PALB2* cDNA with or without variant. Consistently, the same four variants disrupted HR-mediated mRuby integration into the *LMNA* locus. In this assay, the variants exhibited a >90% reduction in HR compared to cells that were complemented with WT *PALB2* cDNA.

Rodrigue et al. (2019) first tested their set of 41 *PALB2* VUS using PARPi sensitivity assays (Table 2). Their assay was set up in HeLa cells in which endogenous *PALB2* was depleted by siRNA treatment. Following transient expression of siRNA-resistant YFP-*PALB2* cDNA, with or without a variant, cells were assayed for PARPi sensitivity. Although no truncating variants were assayed, several benign *PALB2* variants were included (i.e., p.P864S, p.V932M, and p.G998E). As expected, expression of the benign variants rendered cells PARPi resistant, which was comparable to that observed after WT *PALB2* expression (Rodrigue et al., 2019). The threshold for impaired PALB2 function was set based on the PARPi sensitivity observed for cells expressing p.L35P (∼50% survival), which was previously reported to be damaging (Foo et al., 2017). The expression of two *PALB2* variants, p.T1030I and p.W1140G, rendered cells nearly as sensitive as those expressing p.L35P, with survival percentages of 58% and 64%, respectively, while the expression of several other variants (p.P8L, p.K18R, p.R37H, p.H46Y, p.L947F, p.L947S, and p.L1119P) only resulted in a moderate, but still significant sensitivity to PARPi (∼76–86% cell survival). For a more direct assessment of HR competency, the CRISPR-*LMNA* HR assay (Ducy et al., 2019) was used to further characterize the effects of 18 selected *PALB2* variants on HR. Consistently, p.T1030I and p.W1140G exhibited substantially reduced HR (>65% reduction in HR when compared to WT *PALB2*), followed by p.Y28C and p.R37H (60–65% reduction in HR when compared to WT *PALB2*), whereas other variants (p.P8L, p.L947F, p.L947S, and p.G1043A), showed more intermediate phenotypes (40–60% reduction in HR when compared to WT *PALB2*).

In our recent study (Boonen et al., 2019), a large number of *PALB2* truncating variants was included (p.Q60Rfs, p.S172Gfs, p.E230X, p.Y409X, p.L531Cfs, p.Y551X, p.E669Gfs, p.C882Wfs, p.Q988X, p.P1009Lfs, p.W1038X, and p.Y1183X), as well as several variants that were classified as benign by ClinVar (p.Q559R, p.E672Q, p.V858=, p.P864S, and p.G998E) (Table 2). Our functional analysis relied on the stable integration of human *PALB2* cDNA at a safe-harbor locus in *Palb2* knockout (KO) mouse embryonic stem (mES) cells and its subsequent expression from a relatively weak promotor (Boonen et al., 2019). Such a strategy avoids differences in *PALB2* expression following siRNA-mediated knockdown and reduces possible artifacts that may arise from transient overexpression of *PALB2* cDNA. While benign variants had only a moderate or no impact on HR (<20% reduction in HR when compared to WT *PALB2*), all truncating variants strongly impacted HR (>89% reduction in HR when compare to WT *PALB2*). Moreover, 48 *PALB2* VUS were analyzed, of which the expression of 15 VUS (i.e., p.L24S, p.Y28C, p.L35P, p.R37H, p.W912G, p.G937R, p.I944N, p.L947S, p.L961P, p.L972Q, p.T1030I, p.I1037T, p.G1043D, p.L1070P, and p.L1172P), strongly abrogated PALB2 protein function, with HR being decreased by 55–93% in DR-GFP assays. The same variants also resulted in cellular sensitivity to PARPi (Table 2) (Boonen et al., 2019).

## Effect of VUS in PALB2’s CC Domain on the BRCA1-Interaction and HR

Formation of the PALB2-BRCA1/2-RAD51 complex is crucial for delivering RAD51 monomers to RPA-coated ssDNA overhangs and promoting strand invasion during HR (Sy et al., 2009b; Zhang et al., 2009a,b; Jensen et al., 2010). Variants that affect PALB2’s interaction capability with BRCA1 or BRCA2 are therefore predicted to impact HR. Here we first discuss the implications of variants in PALB2’s CC domain (amino acids 9–44) (Figure 1). Initially it was shown by two independent studies that exchange of PALB2’s CC domain residues p.L21, p.Y28, or p.L35 by an alanine (Sy et al., 2009b), or p.L21 or p.L24 by a proline (Zhang et al., 2009a), indeed impaired HR by abolishing the interaction between PALB2 and BRCA1. Consistently, the patient-derived p.L35P missense variant in *PALB2* was more recently shown to impair the interaction with BRCA1, thereby strongly reducing HR (Foo et al., 2017). This variant was taken along by the three recent studies which all confirmed these findings (Boonen et al., 2019; Rodrigue et al., 2019; Wiltshire et al., 2019). A similar defect in HR was observed for p.L24S, which was also attributable to an impairment in the interaction with BRCA1 (Boonen et al., 2019; Wiltshire et al., 2019). Interestingly, cycloheximide chase experiments to monitor protein stability suggested that variants that fail to interact with BRCA1 (p.L24S and p.L35P) enhanced the stability and consequently the levels of PALB2 protein (Wiltshire et al., 2019). Consistent with this result, we and others also detected slightly higher protein levels for variants that failed to interact with BRCA1 (i.e., p.L24S, p.Y28C, and p.L35P) (Foo et al., 2017; Boonen et al., 2019; Wiltshire et al., 2019). As the CC domain regulates PALB2 self-interaction in addition to the interaction with BRCA1, it is possible that an inability of PALB2 to interact with BRCA1 creates a shift toward the formation of PALB2 oligomers (Sy et al., 2009c; Buisson and Masson, 2012). Such complexes may shield PALB2 from ubiquitination-dependent degradation (Orthwein et al., 2015), leading to higher protein levels.

The consistency between the different studies (Boonen et al., 2019; Rodrigue et al., 2019; Wiltshire et al., 2019) was challenged by the analysis of p.R37H, which has previously been shown to represent a variant whose expression only moderately impacts protein function (Foo et al., 2017). p.R37H was shown to reduce HR only by ∼20% (Foo et al., 2017; Wiltshire et al., 2019). Accordingly, the analysis by Foo et al. (2017) showed that p.R37H did not affect the interaction with BRCA1. In contrast, Rodrigue et al. (2019) identified p.R37H as a variant whose expression led to a significant reduction in PALB2 function, both in PARPi sensitivity assays as well as the CRISPR-LMNA HR assay, with 60% reduced activity in the latter assay. However, the mechanism for the reduced functionality was unclear as mammalian two-hybrid assays and laser micro-irradiation experiments suggested that this variant interacted normally with BRCA1 and was recruited to DNA damage sites, respectively. Although we reported a similar impact on HR in DR-GFP assays for this variant (55% reduction in HR when compared to WT *PALB2*) (Boonen et al., 2019), we observed a partial loss of the PALB2-BRCA1 interaction in co-immunoprecipitation experiments, as well as the recruitment of PALB2 to sites of DNA damage induced by laser micro-irradiation (Boonen et al., 2019). Thus, while all four studies consistently show the impact of p.R37H on HR, the discrepancy in the mechanistic explanation warrants further investigation of this particular variant.

## Effect of VUS in PALB2’s WD40 Domain on Protein Stability and HR

In addition to the CC domain, which mediates the interaction with BRCA1, the WD40 domain of PALB2 (amino acids 853–1186) (Figure 1), mediates interactions with other core HR proteins such as BRCA2 and RAD51. In our study, many damaging variants were identified in this functional domain (p.W912G, p.G337R, p.I944N, p.L947S, p.L961P, p.L972Q, p.T1030I, p.I1037T, p.G1043D, p.L1070P, and p.L1172P) (Boonen et al., 2019). Since all these variants exhibited strongly reduced protein expression levels, the effect on the interaction of PALB2 with other HR factors was not examined. Importantly, reverse transcription-quantitative (RT-q)PCR analysis indicated that these variants did not affect expression at the mRNA level (Boonen et al., 2019), suggesting that the low abundance of PALB2 protein is likely the result of protein instability. In contrast, Rodrigue et al. (2019) performed PALB2-BRCA2 co-immunoprecipitation assays for damaging variants in the WD40 domain (p.L947F, p.L947S, p.T1030I, p.G1043A, p.L1119P, and p.W1140G), although they similarly detected lower expression levels for these variants. Not surprisingly, all six variants appeared to impair the interaction with BRCA2. As these variants are scattered throughout the WD40 domain, it seems likely that they represent unstable variants rather than variants that impair specific binding sites for BRCA2. Likewise, Wiltshire and colleagues showed that the p.I944N and p.L1070P variants both decreased the interaction with BRCA2, as well as with BRCA1 (Wiltshire et al., 2019). As the interaction motif for BRCA1 lies in PALB2’s N-terminal CC domain, and not the WD40 domain in which these variants are present, these reduced interactions are more likely the result of reduced PALB2 protein stability. Although we identified several damaging variants in the WD40 domain, only the synthetic missense variant p.A1025R displayed normal expression levels, while having a major impact on HR (82% reduction in HR when compared to WT *PALB2*) (Boonen et al., 2019). These results are in line with the fact that this variant impairs the PALB2-BRCA2 interaction, as shown previously by several studies (Oliver et al., 2009; Rodrigue et al., 2019; Wiltshire et al., 2019).

In addition to the observed protein instability, it has been suggested that mis-localization of the PALB2 protein in the cytoplasm may provide an explanation for the reduced PALB2 functionality observed for a number of variants in the WD40 domain (Park et al., 2014; Boonen et al., 2019; Wiltshire et al., 2019). For instance, for p.I944N, Wiltshire and colleagues showed that this variant prevented nuclear localization of PALB2 and that it is retained in the cytoplasm. They observed a similar mis-localization for p.L1070P, albeit to a lesser extent. Rodrigue et al. (2019) additionally identified p.L947F, p.L947S, p.T1030I, p.G1043A, p.L1119P, and p.W1140G as variants causing mis-localisation of PALB2. All these variants impaired PALB2 recruitment to laser micro-irradiation induced DNA damage, an effect that was also observed for p.Y28C and p.L35P. However, p.Y28C and p.L35P, which both reside in the CC domain, did not negatively impact PALB2’s nuclear localisation. Thus, variants in the WD40 domain that result in PALB2 instability may be signaled for degradation in the cytoplasm, providing an explanation for how such variants could impact PALB2-dependent HR.

## Limitations of Current Assays Used for the Functional Analysis of VUS in *PALB2*

A reasonable number of overlapping VUS was analyzed by the three recent studies (Boonen et al., 2019; Rodrigue et al., 2019; Wiltshire et al., 2019). This allows for a head-to-head comparison of the outcome of the different functional analysis, as well as the important aspects of the different experimental approaches, such as the model cell line, complementation by transient overexpression or stable expression, and the use of KO or knockdown cell lines. Differences therein may explain certain discrepancies in the outcomes, which we discuss below on the basis of several variants that were functionally characterized.

**FIGURE 3:**
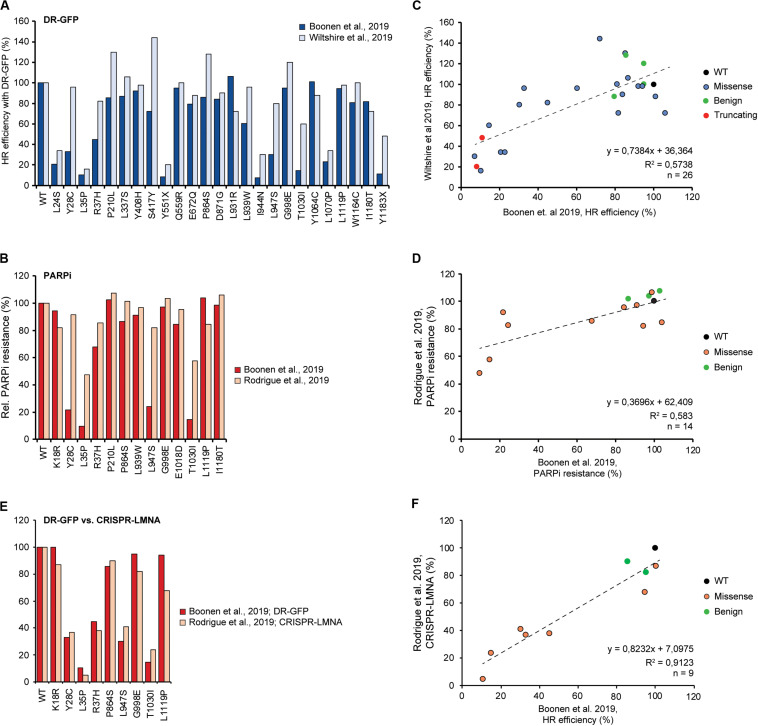
Comparison and correlation between DR-GFP- and PARPi-based HR assays from three different studies. **(A)** Bar graph comparing results from DR-GFP-based functional assays for 26 overlapping *PALB2* variants from studies by us and Wiltshire et al. (2019; Boonen et al., 2019). Mean percentages of GFP-positive cells relative to wild type *PALB2* (WT) are shown, with cells expressing WT *PALB2* being set to 100%. **(B)** Bar graph comparing results from PARPi-based functional assays for 14 overlapping *PALB2* variants from studies by us and Rodrigue et al. (2019; Boonen et al., 2019). Mean percentages of viability relative to WT *PALB2* are shown, with cells expressing WT *PALB2* being set to 100%. **(C)** Scatter plot showing the correlation between the results from our study and Wiltshire et al. (2019) as shown in **(A)** (Boonen et al., 2019). The color of the datapoints corresponds to the different variants/conditions: wild type (black), benign based on ClinVar (green), truncating (red), VUS (blue). **(D)** Scatter plot showing the correlation between the results from our study and Rodrigue et al. (2019) as shown in **(B)** (Boonen et al., 2019). The color of the datapoints corresponds to the different variants/conditions: wild type (black), benign based on ClinVar (green), VUS (orange). **(E)** Bar graph comparing results from DR-GFP- and CRISPR-LMNA-based HR assays for 9 overlapping *PALB2* variants from studies by us and Rodrigue et al. (2019; Boonen et al., 2019). Mean percentages of GFP- or mRuby-positive cells relative to WT *PALB2* are shown as in **(A)**. **(F)** Scatter plot showing the correlation between the results from our study and Rodrigue et al. (2019) as shown in **(E)** (Boonen et al., 2019). The color of the datapoints is as shown in **(D)**.

Overexpression of the *PALB2* cDNA may underestimate the functional effect of some variants. For instance, the FA-associated p.Y1183X *PALB2* variant, which is located three amino acids from the end of the protein, can lead to the expression of a near full-length PALB2 protein. Stable expression of this variant impaired the HR efficiency in mES cells to a similar extent as all other truncating variants positioned throughout the gene (i.e., HR being reduced by 89–94%) (Boonen et al., 2019). However, it is feasible that cDNA-based overexpression of this variant can partially rescue HR. This may have occurred in the study by Wiltshire and colleagues in which p.Y1183X reduced the HR efficiency in *Palb2*-deficient B400 mouse mammary cells by 52%, in comparison to a ∼84% reduction observed for other truncating *PALB2* variants scattered throughout the gene (Wiltshire et al., 2019). Accordingly, Rodrigue et al. (2019) noted that there are indeed differences in expression between variants after transient overexpression. Moreover, they showed that exogenous *PALB2* is greatly overexpressed in comparison to endogenous *PALB2* (Rodrigue et al., 2019). Thus, we may need to take caution when variants are functionally characterized by transient overexpression, as damaging variants may still exhibit residual activity under these conditions. In fact, when we compare other overlapping variants among the three recent studies tested in DR-GFP assays (*n* = 26) (Boonen et al., 2019; Wiltshire et al., 2019) and PARPi sensitivity assays (*n* = 14) (Boonen et al., 2019; Rodrigue et al., 2019), functional defects are almost always smaller when assessed by transient overexpression compared to stable integration and expression (Figures 3A,B). This is particularly striking in the case of variants such as p.Y28C, p.R37H, p.L947S, and p.T1030I, which may still exhibit residual activity. Consequently, this effect may lead to an underestimation of the HR defects that these variants can cause and may explain the fairly low correlation (*R*^2^ = ∼0.58) between results from assays with transient overexpression versus stable integration and expression (Figures 3C,D). However, this hypothesis is contradicted by the very good correlation (*R*^2^ = ∼0.91) (Figures 3E,F) between the effects of overlapping variants in DR-GFP and CRISPR-LMNA HR assays (*n* = 9) (Boonen et al., 2019; Rodrigue et al., 2019), which relied on stable and transient expression of *PALB2*, respectively. Although this result can be explained by a slightly more effective siRNA-based knockdown of endogenous *PALB2* in the U2OS cells used for the CRISPR-LMNA HR assays, it is also possible that stable versus transient expression, in a specific cellular background, impacts the outcome of the functional assays. Further research is therefore needed to resolve these issues.

Similar to transient overexpression, *PALB2* complementation after knockdown of the gene (versus the use of KO cells), could in theory also result in an underestimation of the effects of some variants. This is because the knockdown is often incomplete, resulting in residual expression of wild type *PALB2* in the presence of exogenously expressed *PALB2* carrying a variant. If the *PALB2* variant affects PALB2 protein function, this effect may be obscured by the presence of wild type PALB2 protein. Also, the knockdown efficiency can differ between experiments, resulting in variability in the measured functional effects. On the other hand, with regard to the KO of genes in general, it is possible that cells can undergo adaptions in order to survive. It is possible that such adaptions can influence the functional readout that is used.

As all three recent studies employed a cDNA-based complementation approach (Boonen et al., 2019; Rodrigue et al., 2019; Wiltshire et al., 2019), another disadvantage, specifically when analyzing truncating variants, is the absence of nonsense-mediated mRNA decay. Hypothetically, the expression of a partially functional truncated protein might mask the severe impact on protein function of such variants observed in the presence of nonsense-mediated mRNA decay, which would otherwise abrogate protein expression. A complementation method based on the use of a bacterial artificial chromosome (BAC) that contains the complete gene-of-interest would allow for inclusion of effects originating from nonsense-mediated mRNA decay. This is important, as such processes by themselves may enhance the risk for cancer and constitute an alternative mechanism for reduced protein function.

With regard to the differences in outcome between the three recent studies on *PALB2* VUS (Boonen et al., 2019; Rodrigue et al., 2019; Wiltshire et al., 2019), one may also question whether these may originate from the use of human and mouse model cell lines. For instance, we showed that complementation of *Palb2* KO mES cells with human *PALB2* cDNA resulted in a partial rescue of the HR defect (i.e., ∼68% HR compared to *Trp53* KO cells expressing mouse *Palb2*) (Boonen et al., 2019). Although it cannot be excluded that this is due to different expression levels of ectopic human *PALB2* compared to endogenous mouse *Palb2*, it is also possible that this is due to the limited homology between mouse and human *PALB2* (∼59% identical and 70% similar in protein sequence). Consequently, the functional effect of some variants may be missed and this could affect the reliability of testing human variants in a mouse cell background. Nonetheless, it should be noted that so far damaging missense variants in *PALB2* have only been observed in the well conserved CC and WD40 domains, which both exhibit ∼82.5% identical and ∼91.5% similar protein sequence. This makes it unlikely that *PALB2* variants that have been identified as damaging in these domains in mouse cell-based assays, are not so in a human cell-based setup. Indeed, we have observed similar effects on HR for a number of VUS (p.W912G, p.G937R, p.L947S, p.L961P and G1043D) in human and mouse cell-based assays (Boonen et al., 2019).

## Functional Characterization of VUS in *PALB2* Using Checkpoint Control as a Read-Out

Besides a critical role in promoting HR, several studies have implicated *BRCA1*, *BRCA2* and *PALB2* in DNA-damage-induced checkpoint control (Cotta-Ramusino et al., 2011; Menzel et al., 2011; Simhadri et al., 2019). Consistently, it was shown that G2/M checkpoint maintenance after IR is compromised in *Trp53* KO/*Palb2* KO mES cells, an effect that could be rescued by expressing WT human *PALB2* (Figure 2) (Boonen et al., 2019). Interestingly, *PALB2* variants that show LOF in HR, were unable to maintain an efficient G2/M checkpoint response (p.L35P, p.L961P, p.A1025R, and p.G1043D). The fact that p.L35P and p.A1025R, which are unable to interact with BRCA1 and BRCA2, respectively, were among these variants, suggests that both interactions are key to PALB2’s role in regulating G2/M checkpoint control. Although checkpoint regulation could be a distinct function of *PALB2*, another possibility is that the observed defects in G2/M checkpoint maintenance could stem from defective HR. Given that a defect in HR likely leads to elevated levels of unrepaired DNA breaks, it may seem counterintuitive that G2/M checkpoint maintenance is reduced under these conditions, unless compensatory pathways take over to complete DNA repair and allow for continued progression through the cell cycle. In line with such a scenario, an inverse correlation has been observed between HR activity and a-NHEJ mediated by POLQ (Ceccaldi et al., 2015). This indicates that a-NHEJ may act as a compensatory pathway for PALB2-dependent HR. Indeed, in HR-deficient ovarian cancer cell lines POLQ was selectively upregulated, whereas restoration of HR brought back POLQ expression to normal levels (Ceccaldi et al., 2015). Based on these findings we speculate that when HR is compromised due to PALB2 LOF, activation of a-NHEJ potentially affects G2/M checkpoint maintenance in response to DNA breaks.

## Control of ROS and Replication Stress as Potential Readouts for the Functional Analysis of VUS in *PALB2*

PALB2 has also been reported to play a role in controlling the reactive oxygen species (ROS) levels in human cells (Ma et al., 2012), which may constitute another tumor suppressive function. PALB2 suppresses ROS levels in a manner dependent on its interaction with the ubiquitin ligase KEAP1. KEAP1 functions as a cysteine-rich oxidative stress sensor, which under normal conditions, binds to and targets the antioxidant transcription factor NRF2 for degradation (Ma et al., 2012). As PALB2 bears a highly conserved ETGE-type KEAP1-binding motif (amino acids 88–94), that is identical to that of NRF2, PALB2 can competitively impede the inhibitory KEAP1-NRF2 interaction. Therefore, PALB2 is believed to promote NRF2 accumulation, enhance antioxidant gene expression and reduce the burden of oxidative stress. However, the truncating p.Y551X *PALB2* variant, which has been described to be associated with FA and breast cancer (Xia et al., 2007), still interacts with NRF2 as corroborated by Ma et al. (2012), and consequently should be functional in the regulation of ROS levels. Furthermore, this truncated variant has been shown to be expressed in lymphoblasts of an individual with FA and is apparently not subjected to nonsense RNA-mediated decay (Xia et al., 2007). We therefore infer that the effect of impaired regulation of ROS levels by PALB2 may have no, or only a minor contribution to the development of FA and breast cancer, questioning the value of a more extensive analysis of the effect of VUS in *PALB2* on this process.

Besides playing a key role in HR, *BRCA1, BRCA2* and *RAD51* have also been implicated in replication fork protection and/or the recovery of stalled replication forks, which are processes that are critical for genome stability maintenance and cancer prevention, as well as cancer therapy responses (Schlacher et al., 2011, 2012; Chaudhuri et al., 2016; Sidorova, 2017). Intriguingly, it was recently shown that the interaction between BRCA1 and BARD1 promotes the protection of replication forks and that genetic variants in *BRCA1* that impair this interaction associate with cancer, even though they retain their function in HR (Daza-Martin et al., 2019). A mutational analysis of *BRCA2* revealed that a conserved C-terminal site involved in stabilizing RAD51 filaments, but not in loading RAD51 onto DNA, is essential for replication fork protection, but dispensable for HR. Consistently, the p.S3291A variant in this C-terminal region was shown to impair the protection of stalled replication forks, while leaving HR intact (Schlacher et al., 2011). RAD51, on the other hand, acts during DNA replication to facilitate fork reversal and protects nascent DNA strands from nuclease digestion, thereby promoting the recovery of stalled replication forks (Petermann et al., 2010; Zellweger et al., 2015; Godin et al., 2016). It is plausible that PALB2 exhibits functions at the replication fork that are comparable to those of BRCA1, BRCA2 and/or RAD51. Indeed, it was previously shown that PALB2 mediates replication fork recovery after replication stress in human U2OS cells (Murphy et al., 2014). Corroborating these findings, replication abnormalities, including a decreased/delayed origin firing and replication fork restart, have also been observed in blood lymphocytes heterozygous for the truncating p.L531Cfs *PALB2* variant (Nikkila et al., 2013). How PALB2 mechanistically facilitates these processes is still largely unclear and requires additional research. However, it is feasible that VUS in *PALB2* could have the potential to specifically impair such functions, as has been reported for *BRCA1* and *BRCA2* (Schlacher et al., 2011; Daza-Martin et al., 2019). Potentially, loss of PALB2’s function in replication fork protection and/or recovery may associate with cancer. If so, replication fork maintenance may become another important readout for the functional analysis of *PALB2* VUS.

## *In Silico* Approaches Predicting the Functional Impact of VUS Are Mostly Unreliable

Especially with the vast accumulation of identified VUS (Lek et al., 2016; Karczewski et al., 2019), a variety of *in silico* tools, which are both publicly and commercially available, can aid in the interpretation of VUS in clinical diagnostic settings (Richards et al., 2015). However, the currently available *in silico* tools, such as PolyPhen-2, SIFT, MutationTaster-2, MutationAssessor, CADD and REVEL, often give rise to conflicting results and over- or underestimate the functional impact of a given variant (Grimm et al., 2015; Miosge et al., 2015). A systematic performance comparison between *in silico* prediction tools and functional assays, showed that functional assays substantially outperform every computational method examined, mostly with respect to heightened specificity (Sun et al., 2016). In this study, a panel of 26 different yeast-based complementation assays were used to measure the impact of 179 variants on 22 human disease genes. Remarkably, of the 64 non-disease-associated variants tested, 36% was predicted to be deleterious by PolyPhen, as opposed to only 13% being classified as deleterious by these functional assays (Sun et al., 2016). This high rate of false predictions is in agreement with recent data from us and Rodrigue et al. (2019), showing that *in silico* prediction tools all strongly overpredicted the percentage of deleterious variants in *PALB2* (Boonen et al., 2019). Consistently, these studies observed a poor correlation between results from DR-GFP assays and predictions by CADD (*R*^2^ = 0.08) or REVEL (*R*^2^ = 0.11) (Boonen et al., 2019), and between results from PARPi sensitivity assays and M-CAP (*R*^2^ = 0.33), VEST (*R*^2^ = 0.07) or REVEL (*R*^2^ = 0.27) (Rodrigue et al., 2019). Due to this lack of consistency and poor performance, computational predictions are not considered strong evidence for or against pathogenicity (Richards et al., 2015). Instead, functional assays seem to represent the best strategy for overcoming the VUS challenge, as they currently constitute the strongest evidence for the functional impact of rare variants. Moreover, for genes such as *BRCA1* and *BRCA2*, for which functional assays are more established, a functional read-out such as HR can be used to improve existing computational prediction tools. In a recent study by Hart et al. (2019), the measured HR efficiency for 248 *BRCA1* and 207 *BRCA2* variants was used to recalibrate 40 *in silico* algorithms. Optimized thresholds based on such functional data significantly improved the accuracy of many of these algorithms. However, optimized algorithms for one gene may perform poorly when applied to another gene. This is perhaps not surprising as each functional domain may harbor different sensitivities to the effects of damaging variants, explaining why different gene-specific features are important for the accuracy of *in silico* predictions.

## Perspective on High-Throughput Functional Analysis of *PALB2* Variants

The identification of VUS has increased drastically due to the global build-up in genetic testing (Priestley et al., 2019), leading to major challenges in the clinical management of carriers. To emphasize the vast number of genetic variants that are identified, 4.6 million missense variants have recently been reported in ∼140000 exomes and genomes in the Genome Aggregation Database (gnomAD) (Lek et al., 2016; Karczewski et al., 2019) and 99% of these variants are rare with a minor allele frequency of <0.005 (Starita et al., 2017). Variant interpretation at such a scale, can currently only be addressed with computational prediction tools. However, as mentioned above, the existing tools often provide conflicting results, where functional impact is mostly overpredicted (Sun et al., 2016; Boonen et al., 2019). Thus, the accelerated rate of VUS discovery makes a one-at-a-time approach, or even semi high-throughput methods, for functional analysis infeasible. Furthermore, as these strategies are often time-consuming, the individual in which a variant was found may not be able to take advantage of it in time.

An ambitious goal for the future is that the effect of every possible nucleotide substitution, perhaps initially only in clinically actionable genes (Green et al., 2013; Kalia et al., 2017), is functionally measured using high-throughput assays. For instance, for *PALB2* specifically, as of June 2020, 1612 distinct VUS have been reported in ClinVar. This number already makes a one-at-a-time functional analysis approach extremely challenging. High-throughput assays (i.e., multiplexed assays), aimed to address every nucleotide change in an entire gene in single experiments may provide a solution. Indeed, a saturation CRISPR/Cas9-based editing approach in haploid human HAP1 cells allowed for the assessment of more than 95% of all possible single-nucleotide variants (SNVs) in 13 exons of *BRCA1* that encode for its RING and BRCT domains (Findlay et al., 2018). Importantly, this setup allowed for the functional analysis of variants in their endogenous genomic context and using cell survival as a read-out, the effect of nearly 4000 single-nucleotide variants corroborated established assessments on protein function. Furthermore, a multiplex homology-directed repair assay, which relied on stable integration of a *BRCA1* cDNA variant library, enabled the functional characterization of 1056 missense variants in the first 192 residues of *BRCA1* (Starita et al., 2018). We expect that in the near future such assays will be extended to analyzing variants in genes such as *PALB2*, ultimately leading to the development of a variant map that shows the impact of all possible *PALB2* variants on HR.

In addition to examining cell survival and HR for *PALB2* in a high-throughput setup, another more general readout might be to measure the steady-state protein abundance. Recent results from functional assays have shown that variants in PALB2’s WD40 domain tend to destabilize PALB2 (Boonen et al., 2019), a mechanism of protein inactivation that is in agreement with studies showing that ∼75% of pathogenic variation is thought to disrupt thermodynamic stability and alter protein levels (Yue et al., 2005; Redler et al., 2016; Matreyek et al., 2018). Therefore, high-throughput assessment of PALB2 variant protein abundance, by employing techniques such as VAMP-seq (Matreyek et al., 2018) or Stable-seq (Kim et al., 2013), may also prove to be highly suitable for detecting *PALB2* variants that affect protein function. Nonetheless, although such high-throughput assays provide much potential for interpreting the large number of VUS that are being identified, it should also be noted that developing variant libraries, optimizing experimental setups, and analyzing the large amount of sequencing data, can still be prohibitively time and resource intensive.

## Toward the Functional Analysis of *PALB2* VUS in RNA Splicing

It is important to note that all functional studies on VUS in *PALB2* discussed in this review (Park et al., 2014; Foo et al., 2017; Boonen et al., 2019; Rodrigue et al., 2019; Wiltshire et al., 2019), were based on expression of *PALB2* cDNAs and are therefore not suitable to assess the functional impact of *PALB2* variants that affect RNA splicing. *In silico* splice site prediction tools can predict the effect of variants on potential splice sites relatively well (Richards et al., 2015), but they do not provide conclusive evidence for altered splicing. One option to assess the effect of variants on splicing, is to use a minigene construct that contains a genomic segment encompassing the variant along with flanking intronic sequences (Gaildrat et al., 2010). After transient transfection of the construct into human cells, the transcripts from the minigene can easily be analyzed and compared to transcripts derived from a wild type construct. Although these assays can be carried out in many cell types and are fairly simple and fast, disadvantages are that variants are not measured in the context of a complete gene and that these assays do not permit downstream functional analysis. This is of course important since some splice variants can result in the expression of a transcript that may be (partially) functional. For instance, several exons in *PALB2* (exons 1, 2, 4, 6, 7, 9, 10, and 11–12 combined) can be skipped due to splice site variants and still result in an in-frame transcript (Lopez-Perolio et al., 2019). Such transcripts may still express an isoform of *PALB2* with an entire exon deleted, yet retain partial protein function. An example is the c.2586+1G > A (r.2515_2586del; p.T839_K862del) *PALB2* variant, which leads to an in-frame skip of exon 6. This variant appears to be a hypomorphic variant that still interacts with BRCA2 and, when overexpressed, still enables RAD51 foci formation (Byrd et al., 2016). Additional research will be required to establish the functionality of other exon-skip variants in *PALB2.*

As of June 2020, 70 unique *PALB2* splice variants have been reported in ClinVar (involving canonical splice sites), the majority of which is classified as pathogenic or likely pathogenic. Generally, mRNA transcript and protein expression analysis combined with functional assays, may be needed to provide insight into the effect of variants in *PALB2* that are predicted to impact RNA splicing. Possibly, one could complement *PALB2* KO cells containing DR-GFP with a BAC containing the full length human *PALB2* gene. Such a method has previously been described for *BRCA1* and *BRCA2* (Kuznetsov et al., 2008; Chang et al., 2009; Mesman et al., 2018, 2020) and would allow for the introduction and functional analysis of splice variants in coding and non-coding regions, further improving their classification.

## Toward Estimating Cancer Risk Associated With VUS in *PALB2*

Functional assays may aid in the classification of rare *PALB2* VUS, yet a major challenge will be to translate effects on PALB2 protein function into estimates for cancer risk. Recent studies on *BRCA2* have shown that pathogenic variants that confer high risk for breast and ovarian cancer completely abrogate BRCA2-mediated HR, whereas variants that result in a reduction of 50% in HR, i.e., hypomorphic variants, may only be associated with a moderate risk for breast cancer (Odds ratio ∼2.5) (Shimelis et al., 2017; Mesman et al., 2019). With regard to *PALB2*, truncating variants have been associated with an odds ratio of 7.46 (95% CI, 5.12–11.19) (Couch et al., 2017), whereas the frequently occurring p.L939W missense variant has been associated with an odds ratio of 1.05 (95% CI, 0.83–1.32) (Southey et al., 2016), which is in agreement with recent data from Rodrigue et al. (2019) and Wiltshire et al. (2019) showing that this variant does not impact the HR efficiency (∼4% reduction in HR when compared to WT). In contrast, results from us and Park et al. (2014) showed that this variant did impair HR to some degree (40 and 15% reduction in HR when compared to WT, respectively) (Boonen et al., 2019). This may suggest that such a decrease in HR, may not considerably increase the risk for breast cancer. Future functional characterization of additional *PALB2* VUS, in combination with data from large case-control association studies, should allow for more conclusive correlations of odds ratios with HR efficiencies for *PALB2*, either for specific variants that occur frequently, or for variants as a group (i.e., damaging variants). Under the assumption that variants with similar levels of HR functionality confer the same level of cancer risk, so called burden-type of association analyses can be performed in large case-control studies, in which either genetic or clinical information of multiple variants, or joint frequencies of individual variants with similar HR levels will be pooled. Nonetheless, the fact that roles other than in HR (i.e., in replication fork stability/recovery) for all three major breast cancer susceptibility genes (*BRCA1*, *BRCA2* and *PALB2*) have been described (Schlacher et al., 2011; Murphy et al., 2014; Chaudhuri et al., 2016; Daza-Martin et al., 2019), complicates the interpretation of VUS in these genes and their association with cancer risk. It should be noted, however, that only a few variants in *BRCA1* and *BRCA2*, have recently been implicated in the protection of replication forks, while having no impact on HR (Schlacher et al., 2011; Daza-Martin et al., 2019). To our knowledge, no such variants have yet been reported for *PALB2*. Although these *BRCA1* and *BRCA2* variants appear to associate with cancer, their exact risk needs to be further established.

## The Use of Functional Assays for Predicting Therapy Response

Although healthy cells can often repair DNA damage by making use of their full repertoire of DNA repair mechanism, cells exhibiting deficiency in HR due to the presence of *PALB2* LOF variants, become more reliable on alternative DNA repair mechanisms to survive and proliferate. Therefore, conventional treatment strategies (especially for HR-deficient tumors) have been developed to force DNA damage-induced cell death through synthetic lethal interactions. It is now well established that cancers that exhibit pathogenic variants in *BRCA1* or *BRCA2* respond well to treatment with PARPi (Bryant et al., 2005; Farmer et al., 2005), a therapeutic strategy that has emerged for *BRCA1-* and *BRCA2*-mutated breast and ovarian tumors (Audeh et al., 2010; Tutt et al., 2010; Lord and Ashworth, 2017). Consequently, it is of great importance to identify deleterious *PALB2* VUS that lead to HR deficiency and for which corresponding tumors may similarly respond to PARPi-based therapy.

With regard to the studies that functionally analyzed VUS in *PALB2* (Park et al., 2014; Foo et al., 2017; Boonen et al., 2019; Rodrigue et al., 2019; Wiltshire et al., 2019), it is clear that within each study, the HR efficiency correlated extremely well with PARPi sensitivity, exhibiting a strong positive correlation in mES cells (*R*^2^ = 0.804) (Boonen et al., 2019) and human cell lines (*R*^2^ = 0.68) (Rodrigue et al., 2019). Similar results were obtained for sensitivity assays with cisplatin (*R*^2^ = 0.8313) (Boonen et al., 2019; Wiltshire et al., 2019), a commonly-used chemotherapeutic for many cancers, including breast and ovarian cancer. Similar to that in many *BRCA1*- and *BRCA2*-associated tumors (Maxwell et al., 2017; Riaz et al., 2017), many *PALB2*-associated breast cancers (i.e., 67%) show loss of the *PALB2* wild type allele via acquired pathogenic somatic variants, or via loss-of-heterozygosity (LOH) (Lee et al., 2018; Li et al., 2019). Such *PALB2*-null cancers all exhibited HR deficiency, with some tumors even showing HR deficiency while the wild type allele was retained (Lee et al., 2018; Li et al., 2019), suggesting that also alternative mechanisms for PALB2 LOF can be in play. With results from such studies in mind, findings from functional assays that show which VUS are damaging or functional, may prove to be valuable for predicting platinum- and/or PARPi-based therapy response in cancer patients that carry *PALB2* variants that abrogate HR.

## Concluding Remarks

Due to the accelerating pace by which genetic variants in *PALB2* are discovered, there is a strong need to determine which variants actually associate with disease causation. The combined effort to functionally characterize 155 *PALB2* genetic variants, for which clinical significance is unknown, represents a milestone in the reclassification of these variants. Classification of VUS to a category with a defined clinical significance is of great importance to carriers of a pathogenic variant. This will allow them to make an informed decision on how to manage their cancer risk, including increased surveillance or risk reducing surgery to reduce cancer incidence and/or offering testing of relatives at risk. Counselees carrying non-pathogenic variants may be discharged from intensive follow-up and avoid unnecessary risk-reducing surgery (Plon et al., 2008).

In this review, we have provided head-to-head comparisons of the different assays that were used for the functional characterization of variants in *PALB2*. These analyses are an important starting point for the identification of variants that impact its major tumor suppressive function, which most likely is to be attributed to its role in HR, and whose defects correlate with significantly increased cancer risk. Although these assays were able to consistently determine effects of several variants on PALB2’s function during HR, some differences in PALB2 function were also observed (Figure 3), which may be attributed to the type of cDNA-based complementation approach being used. With regard to functional assays being used as clinical diagnostic tools, it is essential to combine results from functional assays that have been obtained by employing different experimental strategies (Richards et al., 2015; Brnich et al., 2019; Monteiro et al., 2020). Moreover, most functional assays use HR as a read-out. However, if PALB2’s role in checkpoint control, the regulation of cellular ROS levels and/or the maintenance of replication fork integrity may contribute to its tumor suppressive function as well, expanding the different read-outs of functional assays to cover these aspects of PALB2 function will be a must. Generally, these assays should also include the possibility of a combined mRNA and protein expression analysis in order to provide insight into the effect of variants in coding and non-coding regions of *PALB2* that are predicted to affect RNA splicing, further improving their classification.

Until more conclusive correlations between the level of impairment of protein function and associated cancer risk have been established, results from functional assays should be implemented with care when making a clinical assertion with regard to associated cancer risk and targeted therapies. In light of the increasing number of *PALB2* variants that will undoubtedly be identified in the future, this information will ultimately be crucial for clinical geneticists in selecting the appropriate strategy for clinical management of carriers of (rare) variants in *PALB2*.

## Author Contributions

RB, MV, and HA conducted the literature research and wrote the manuscript. All authors contributed to the article and approved the submitted version.

## Conflict of Interest

The authors declare that the research was conducted in the absence of any commercial or financial relationships that could be construed as a potential conflict of interest.
